# Genome-wide identification of the WRKY gene family in *Camellia oleifera* and expression analysis under phosphorus deficiency

**DOI:** 10.3389/fpls.2023.1082496

**Published:** 2023-05-25

**Authors:** Wenjuan Su, Zengliang Zhou, Jin Zeng, Ruilan Cao, Yunyu Zhang, Dongnan Hu, Juan Liu

**Affiliations:** ^1^ Jiangxi Provincial Key Laboratory of Silviculture, College of Forestry, Jiangxi Agricultural University, Nanchang, China; ^2^ Jiangxi Provincial Key Laboratory of Camellia Germplasm Conservation and Utilization, Jiangxi Academy of Forestry, Nanchang, China

**Keywords:** *Camellia oleifera*, Phosphorus deficiency, P-efficient variety, expression profile, cultivar specificity

## Abstract

*Camellia oleifera* Abel. is an economically important woody edible-oil species that is mainly cultivated in hilly areas of South China. The phosphorus (P) deficiency in the acidic soils poses severe challenges for the growth and productivity of *C. oleifera*. WRKY transcription factors (TFs) have been proven to play important roles in biological processes and plant responses to various biotic/abiotic stresses, including P deficiency tolerance. In this study, 89 WRKY proteins with conserved domain were identified from the *C. oleifera* diploid genome and divided into three groups, with group II further classified into five subgroups based on the phylogenetic relationships. WRKY variants and mutations were detected in the gene structure and conserved motifs of *CoWRKYs*. Segmental duplication events were considered as the primary driver in the expanding process of WRKY gene family in *C. oleifera*. Based on transcriptomic analysis of two *C. oleifera* varieties characterized with different P deficiency tolerances, 32 *CoWRKY* genes exhibited divergent expression patterns in response to P deficiency stress. qRT-PCR analysis demonstrated that *CoWRKY11*, -*14*, *-20*, -*29* and -*56* had higher positive impact on P-efficient CL40 variety compared with P-inefficient CL3 variety. Similar expression trends of these *CoWRKY* genes were further observed under P deficiency with longer treatment period of 120d. The result indicated the expression sensitivity of *CoWRKYs* on the P-efficient variety and the *C. oleifera* cultivar specificity on the P deficiency tolerance. Tissue expression difference showed *CoWRKYs* may play a crucial role in the transportation and recycling P in leaves by affecting diverse metabolic pathways. The available evidences in the study conclusively shed light on the evolution of the *CoWRKY* genes in *C. oleifera* genome and provided a valuable resource for further investigation of functional characterization of *WRKY* genes involved to enhance the P deficiency tolerance in *C. oleifera.*

## Introduction

Phosphorus (P), as a fundamental constituent of macromolecules substances, is essential for plant growth and development. Soil total P is relatively plentiful, but most P in soil is mainly fixed in the form of calcium P (alkaline soil), iron P (acidic soils), aluminum P (acidic soils), or organic P (Schachtman et al., 1998). Soluble P, which can be absorbed by plant roots, has low availability and poor mobility in most soils (Shen et al., 2011; Wu, 2013). Thus, P limitation have triggered a variety of plant adaptive strategies to reduce P utilization and/or enhance P acquisition and recycling (Fang et al., 2009). Among the molecular responses to cope with P deficiency, transcription factors have been demonstrated to play essential roles in launching and regulating adaptive mechanisms (Yang and Finnegan, 2010).

The WRKY transcription factors are among the largest families of transcriptional regulators in plants, and participate in a variety of plant biological processes through a set of signaling networks, including plant growth, development, metabolism, and stress responses (Eulgem et al., 2000). WRKY proteins comprise of at least one DNA binding domain of about 60 amino acids residues, a highly conserved WRKYGQK heptapeptide at the N-terminus, and a C2H2 or C2HC zinc finger motif at the C-terminus (Eulgem et al., 2000). WRKY transcriptional factors often modulate gene expression by preferentially recognizing and binding to the W-box in the promoter region of target genes, and participate in the response of plants to various environmental stresses and the regulation of plant growth and development.

Over the past decade, a large number of studies on functional analysis of WRKY genes in many plants have focused on the regulatory function of WRKY transcription factors in plant stress defense networks. Furthermore, with the increasing development of high throughput sequencing technology and bioinformatics, an increasing number of instances has been accumulated to demonstrate that WRKY plays crucial roles in plant responses to P deficiency. For example, in Arabidopsis, *AtWRKY75* was induced by P deficiency and its expression in roots, leaves and flowers was up-regulated by P deficiency, indicating that this member of the WRKY gene family played an important role in promoting phosphorus transport and plant growth and development (Devaiah et al., 2007). In addition, *AtWRKY18* and *AtWRKY40* were also strongly induced in roots at the early stage of P deficiency treatment (Lin et al., 2011). In rice, overexpression of *OsWRKY74* can improve the growth of roots and aboveground under P deficiency treatment (Dai et al., 2016). The overexpression of *TaWRKY72b-1* in wheat (*Triticum aestivum*) can improve the dry weight, P accumulation per plant and P utilization efficiency under P deficiency conditions (Miao et al., 2009).

**Figure 1:**
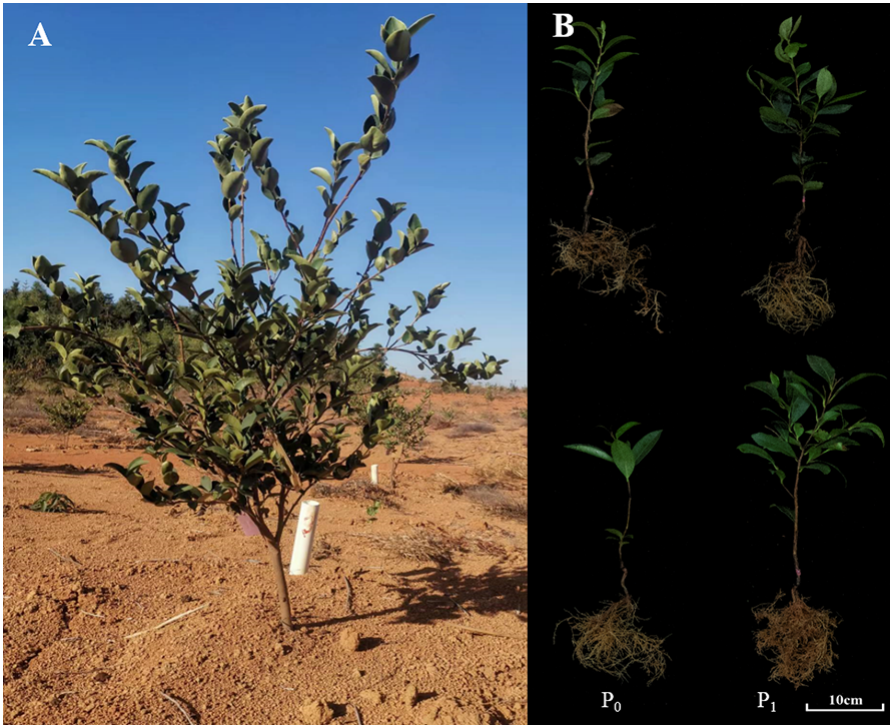
The *C oleifera* tree and its seedlings under different P treatments. **(A)** three-year-old *C oleifera* tree in P deficient acidic soils; **(B)** the *C oleifera* seedlings of P-efficient (in the upper) and P-inefficient varieties (in the bottom) at P deficient (P_0_) and sufficient (P_1_) supplies.

*Camellia oleifera* Abel., ranked as the world’s four major woody oil species, has been widely used as an edible oil, lubricant, and in cosmetics. Because of high content of unsaturated fatty acid (more than 80%), the seed *C. oleifera* is well known as the “Oriental Olive Oil” (Li et al., 2016). In China, *C. oleifera* is the dominant woody oilseed crop with long cultivation history (over 2300 years) and often planted in the low mountains and hilly areas of the Yangtze River Basin and South China. The soil in the extensive planting areas is mainly acid soils with P deficiency (**Figure 1A**) (Li et al., 2019). The shortage of P has posed a challenge to the growth and high yield of *C. oleifera* cultivation (Wu et al., 2019). Therefore, it is of great significance to explore the molecular underlying mechanism of P absorption and utilization in *C. oleifera* and to speed up the selection P-efficient varieties in *C. oleifera.*

Because of polyploidization (2n=6x=90) and high self-incompatibility of *C. oleifera*, genomic sequencing and assembly had been challenged for a long time. The high-quality reference genome of wild diploid *C. oleifera* provides an opportunity to identify gene families in the whole genome level (Lin et al., 2022). In this present study, we performed a comprehensive bioinformative analyses of *CoWRKY* genes using genome-wide data and explored expression traits of *CoWRKYs* in response to P deficiency. The results will provide candidate genes and valuable information for further functional investigations of the *CoWRKYs* on the efficient utilization of P fertilizer in *C. oleifera*.

## Materials and methods

### Plant materials and P deficiency treatments

To investigate the responses of *CoWRKY* genes to extreme P deficiency, two *C. oleifera* varieties with different P efficiency were selected based on the differences in root to shoot weight ratio (R/S), root areas (RA) and total P utilization efficiency (PUE) under the P deficient availability (**Figure 1B**). *C. oleifera* variety of CL40 with higher R/S, RA and PUE, was classified as P efficiency variety, while the variety of CL3 with poor performance on these features was defined as P inefficiency variety (Zeng et al., 2021). All two-year-old potted seedlings from nursery garden were planted in plastic containers (diameter and depth of 20.5 and 17.0 cm, respectively) filled with river sand. The experiments were conducted in a greenhouse at the Science and Technology Park of Jiangxi Agricultural University, Nanchang, China (28^°^450 N, 115^°^490 E). In this study, extremely low P (0 mM) was used as P deficiency and the sufficient P availability (1 mM) was set as the control treatment (Zeng et al., 2022). Hoagland nutrient solution was as follows: 2.0 mM KNO_3_, 2.0 mM Ca(NO_3_)_2_, 2.0 mM MgSO_4_, 50 μM H_3_BO_3_, 50 μM MnSO_4_, 15 μM ZnSO_4_, 2.0 μM CuSO_4_, 14 μM Na_2_MoO_4_, and 50 μM Fe-Na_2_EDTA. KH_2_PO_4_ was employed as P source. KCl was added to ensure identical potassium concentrations under the P-deficiency treatment. In order to investigate the performance of *CoWRKY* genes on P deficiency, two experiments with different treatment durations were carried out in the current study. (i) The first experiment was performed in April 2020, when sufficient nutrients are needed for rapid growth of *C. oleifera*. All similarly developed plants were treated with two levels of P availability. The young leaves were collected at 0, 12, 24, 48, 72 and 96 h after treatment. The time point of 0 h was set as a control. (ii) In the second experiment, all uniformly developed plants were treated with a period of 120 d since March 2019. The corresponding Hoagland nutrient solution was irrigated at a rate of 200 mL for each plant every 12 days. The young leaves were collected at 30 d, 60 d and 120 d. Each treatment had three replicates. In addition, roots, stems, leaves, flowers, young fruits, and ripe kernels from *C. oleifera* trees were collected for gene expression analysis. All samples were rapidly frozen in liquid nitrogen, and then stored at the −80˚C refrigerator.

### Genome-wide identification of the WRKY genes in *C. oleifera*

The diploid *C. oleifera* var.”*Nanyongensis*” genome was obtained from GitHub (https://github.com/Hengfu-Yin/CON_genome_data ). Hidden Markov Model (HMM) profile of the WRKY family domain PF03106 was downloaded from the Pfam database (http://pfam.xfam.org/). WRKY transcription factor was identified using HMMER 3.0 software. The default parameters were set and the expectation (E) value was less than 0.01. Moreover, WRKY protein sequence of *A. thaliana* was downloaded from the Information Resource Website of *A. thaliana* (http://www.arabidopsis.org/). All sequences were subjected to BLASTp search, with the criteria of an e value of e^-10^ and minimum amino acid identify of 40%. SMARAT (http://smart.embl-heidelberg.de/), pfam (http://pfam.xfam.org/), and CCD (https://www.ncbi.nlm.nih.gov/cdd/) were used to validate all identified candidate genes with WRKY domain. All 89 possible *CoWRKYs* are listed in **Table S1**.

The physicochemical parameters and chromosomal location analysis of the candidate *CoWRKYs* genes were further investigated. The primary structure, molecular weight (MW), and theoretical isoelectric points (pIs) of CoWRKYs proteins were predicted using ProtParam (http://web.expasy.org/protparam). The subcellular localization was predicted using CELLO v.2.5 (http://cello.life.nctu.edu.tw/).

### Classification and phylogenetic analysis of CoWRKY proteins

All WRKY protein sequences in *A. thaliana* and *C. oleifera* were subjected to further phylogentic analysis. Multiple alignments of WRKY proteins were carried out using Clustal X2.1 program with default parameters (Larkin et al., 2007) and results were colored using GeneDoc (Version 2.7.0). The phylogenetic tree using conserved region of WRKY proteins was constructed by neighbor-joining (NJ) method of MEGA 7.0 with 1000 bootstrap replications (Kumar et al., 2016). Based on the NJ tree, *CoWRKYs* were classified based on a homology analysis using the *AtWRKYs*. The phylogenetic tree was annotated by the online software iTOL v6 (https://itol.embl.de/).

### The *cis*-acting elements in *CoWRKYs* promoter regions

To identify cis-regulatory elements in *CoWRKY* genes, the upstream sequence (2000 bp) of the promoter regions was obtained by comparing coding sequence with the genome of *C. oleifera*. *Cis*-acting elements were searched and analyzed using the online PlantCARE database (http://bioinformatics.psb.ugent.be/webtools/plantcare/html/) and visualized with TBtools (Lescot, 2002; Chen et al., 2020).

### Chromosomal distribution and duplication of *CoWRKY* genes

The chromosomal distribution of WRKY genes in *C. oleifera* were determined from the annotation file (GFF3) and visualized by mapchart (Version 7.0). Multiple Collinear Scanning toolkits (MCScanX) were employed for gene duplication and synteny analysis, with the default parameters, and visualized by Circos.

### Expression patterns of *CoWRKYs* based on transcriptomes

Expression pattern of *CoWRKY* genes under P deficiency treatment was generated based on P deficiency stress transcriptome (PRJNA831290). P deficiency treatments (0 mM) and control treatments (1 mM) were set up. The leaves of P-efficient variety of CL40 and P-inefficient variety of CL3 were collected on the 30th day and 90th day after P deficiency treatment (Zeng et al., 2021). Transcripts per kilobase million (TPM) values of all candidate *CoWRKYs* for each transcriptome data were evaluated to investigate the abundance of *C. oleifera* WRKY transcripts. A heat map was generated by TBtools software based on the value of log_2_ (TPM + 0.01).

### RNA extraction and qRT-PCR

Total RNA was extracted using the RNA Isolation Kit (DP441, Tiangen Biotech, China) following the manufacture’s instruction. The purity and concentration of the isolated RNA samples were examined on 1.0% (w/v) agarose gels and in a biophotometer (D30, Eppendorf, Germany). Primers for quantitative real-time reverse transcription PCR (qRT-PCR) were designed using Primer 3.0 (www.primer3plus.com/cgi-bin/dev/primer3plus.cgi). All primer sequences were provided in **Table S2**. The first-strand cDNA was synthesized using the iScriptTM cDNA Synthesis Kit (BIO-RAD, China). SYBR Green reagents were used to detect the target sequence. Each PCR mixture (20 µL) contained 1 µL of cDNA, 10 µL of iTaqTM Universal SYBR Green Supermix (Model: 1725121), 1 µL of each primer (10 µM), and 7 µL of ddH_2_O. The qRT-PCR were performed using the following program: 95°C for 2 min and 40 cycles of 95°C for 10 s, 60°C for 30 s, and 72°C for 30 s. Housekeeping gene *EF1a1* was adopted (Song et al., 2014). Each reaction was analyzed in triplicate and the 2^-ΔΔCT^ method was used to analyze the expression patterns of *CoWRKYs*. All experiments were performed with three independent technical replicates.

## Results

### Identification of the *WRKY* genes in *C. oleifera*

In this study, we extracted the WRKY genes from the *C. oleifera* genome using the BLASTP-HMMER methods, and identified 89 *CoWRKY* genes from *C. oleifera* after removing the redundant sequences. All *CoWRKY* gene members were further confirmed based on Pfam, SMART, and CCD databases (**Table S1**). Protein lengths of CoWRKY ranged from 151 amino acid (aa) in *CoWRKY77* to 758 aa in *CoWRKY62*. The predicted molecular weight varied from 16.70 kDa to 81.99 kDa. The theoretical isoelectric points (PIs) of the proteins ranged from 5.01 to 10.33, among which 46 *CoWRKYs* were acidic proteins with pI value <7.0, and the remaining 43 proteins were basic proteins. The prediction of subcellular localization analysis showed that 89 *CoWRKY* proteins were located in the nucleus.

### Classification and phylogenetic analysis of CoWRKY proteins

**Figure 2:**
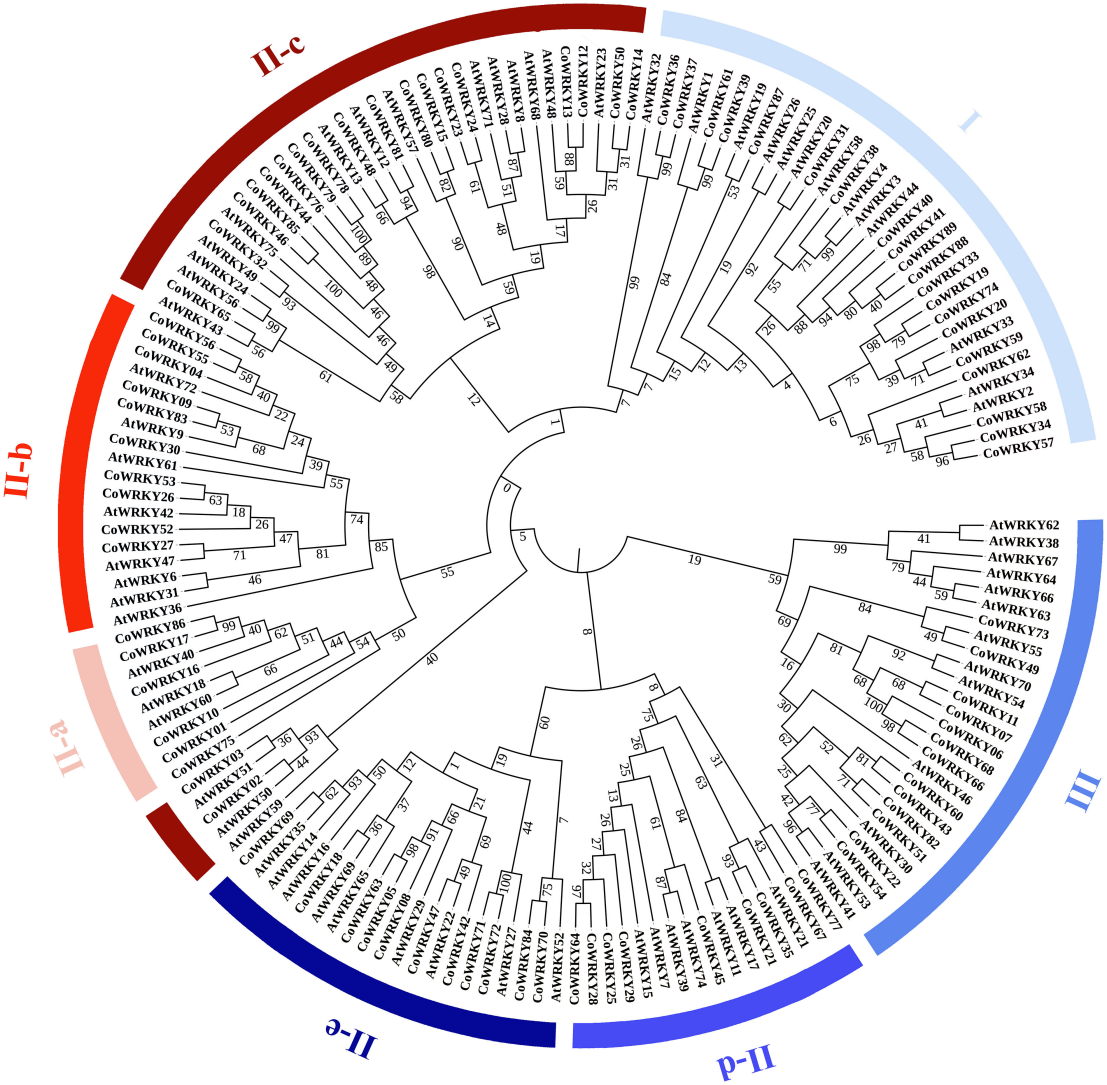
Phylogenetic tree using conserved domain of WRKY genes among *C. oleifera* and *A. thaliana*. The groups (I, II, and III) and subgroups (II-a, b, c, d and e) were distinguished in different colors. Bootstrap values were labelled in the branch of phylogenetic tree.

7Based on the multiple-aligned conserved region of WRKY domain sequences for Arabidopsis and *C. oleifera*, an unrooted Neighbor-Joining phylogenetic tree was established with 1000 bootstrap replications (**Figure 2**). According to the classification of *AtWRKYs* based on Eulgem et al. (2000) and primary amino acid structure feature of *WRKY*, the phylogenetic analysis showed that all CoWRKY proteins were clustered into three major groups, corresponding to Group I, II, and III. Group II accounted for the largest part with *56 CoWRKY* members, followed by Group I with 20 members. However, the number of CoWRKY proteins in Group III was the lowest, including 13 members. In Group II, CoWRKY proteins could be further divided into five subgroups. Subgroup II-a, II-b, II-c, II-d, and II-e contained 6, 10, 20, 9, and 11 members, respectively. The WRKYs proteins is typically characterized by the 60-amino acid WRKY domain, which contains the highly conserved heptapeptide WRKYGQK at the N-terminus followed by a C2H2- or C2HC-type zinc finger motif. Therefore, on one hand, CoWRKY proteins from Group I and III were found to contain two conserved WRKY domains at the N and C termini, while those proteins from Group II usually contained one WRKY domain (**Figure S1**). On the other hand, C2H2-type zinc finger motif was found in the WRKY proteins from Group I and II, while C2HC-type were found in group III. In addition, some exceptions with variants WRKYGQK were found in CoWRKY proteins. For instance, *CoWRKY87* from Group I lost the conserved heptapeptide. *CoWRKY2* and -*3* (from Group II-c) had WRKYGQK variations (WRKYGKK), while *CoWRKY67* (from Group II-d), -*75* (from Group II-a) and -*77* (from Group II-d) lost their zinc finger structure in the C-terminus conserved domain.

### The *cis*-acting elements in *CoWRKYs* promoter regions

**Figure 3:**
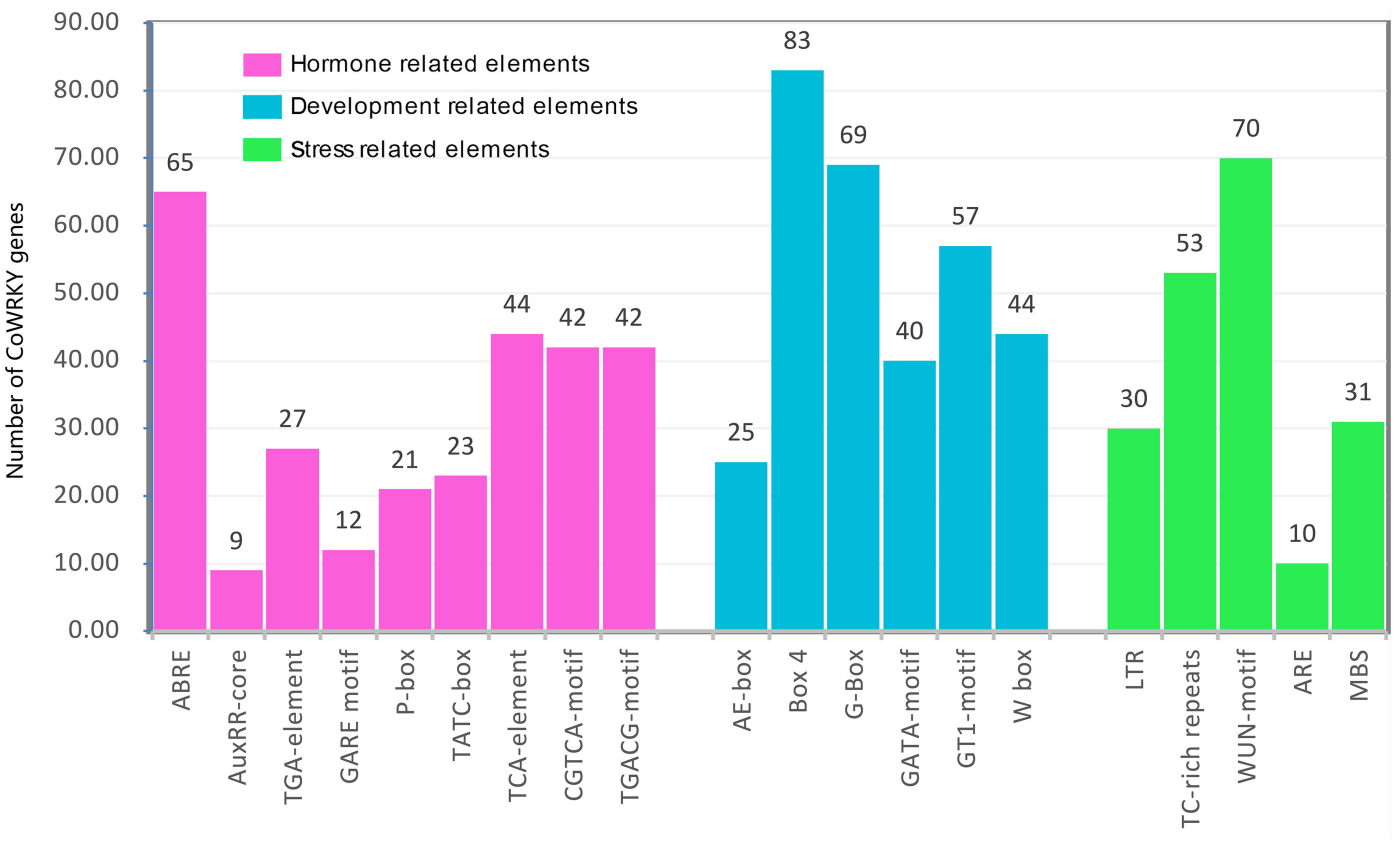
The number of *CoWRKY* genes containing diverse cis-acting elements. X-axis and Y-axis represented the type of *cis*-acting elements and the number of WRKY gene family members, respectively.

The *cis*-acting regulatory elements are essential for the gene promoter transcriptional activity to control the gene expression. In this study, the 2000 bp 5’-upstream promoter regions were employed to predict the *cis*-acting elements using PlantCARE. As a result, a series of hormone-, development- and stress-related *cis*-acting elements were found (**Figure 3**). The hormone-related *cis*-acting elements, including abscisic acid responsiveness (ABRE), auxin responsiveness (AuxRR-core and TGAelement), gibberellin-responsive elements (GARE-motif, P-box, and TATC-box), salicylic acid-responsive (TCA-element), and MeJA-responsive (CGTCA-motif and TGACGmotif), were widely present in the promoter region. Among them, 196 ABRE *cis*-elements were distributed on the promoter region of 65 *CoWRKY* genes, and 32 promoters had at least three ABRE *cis*-elements (**Table S3**). Six development-related *cis*-acting elements, including light-related responsiveness (AE-box, Box 4, G-box, GATA-motif and GT1-motif) and WRKY TF binding element (W-box), were found in the promoter region of *CoWRKYs*. Five stress-related cis-elements, including anaerobic element (ARE), drought stress-responsive element (MBS), defense and stress-responsive element (TC-rich), wound-responsive element (WUN-motif), low-temperature stress responsive element (LTR), were detected. ARE was the most common element in 70 *CoWRKY* promoters, which was a *cis*-acting regulatory element essential for anaerobic induction.

### Chromosomal distribution and gene duplication of *CoWRKY* family

**Figure 4:**
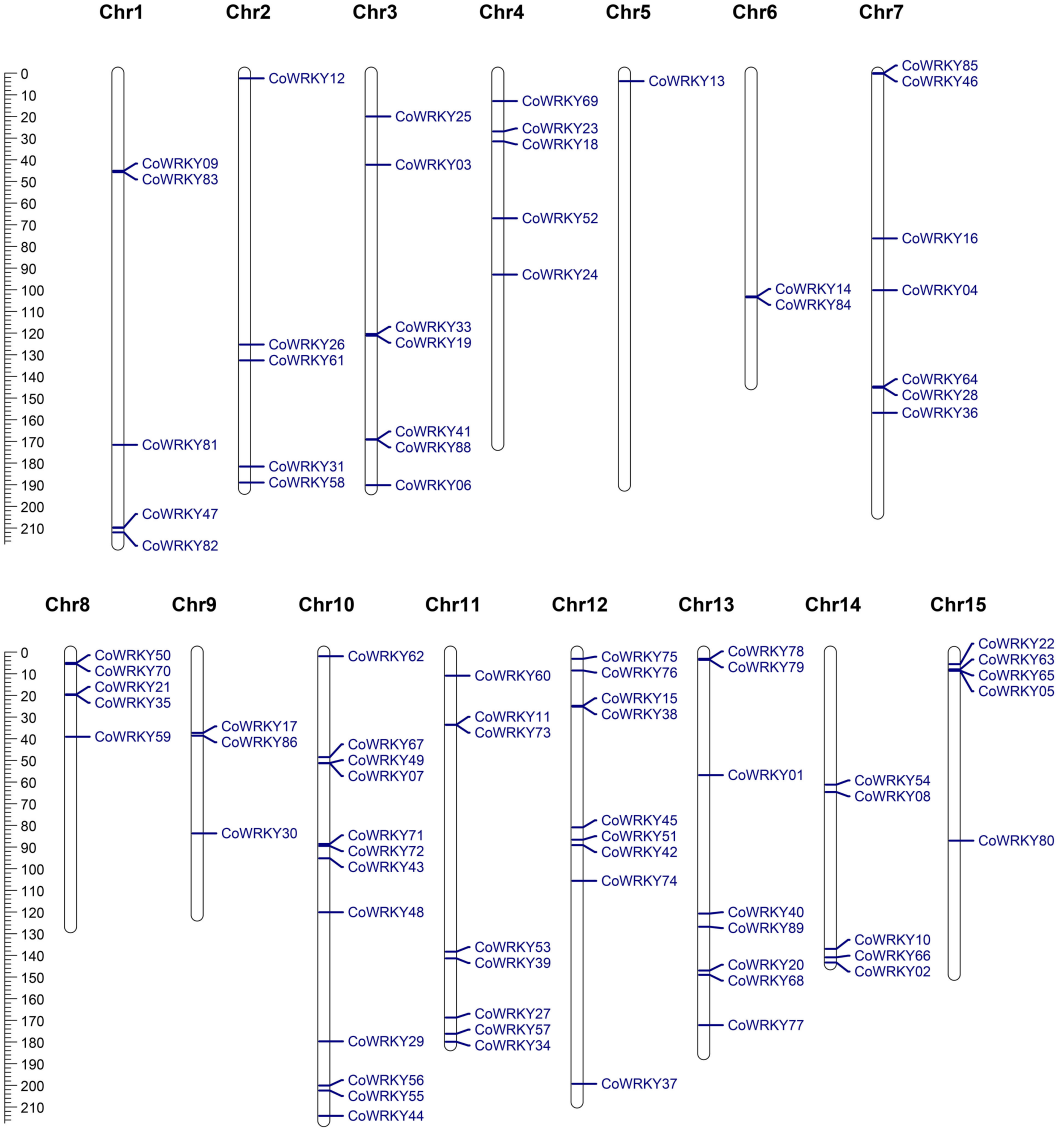
Physical mapping of *CoWRKY* genes on chromosome. The chromosome numbers were indicated at the top of each chromosome. The scale was in megabases (Mb).

Chromosomal distribution analysis revealed that a total of 87 *CoWRKY* genes were unevenly distributed in 15 chromosomes (**Figure 4**). Every chromosome was found to contain at least one *CoWRKY* gene, but their distribution was heterogeneous. The chromosome 10 had the maximum numbers of *CoWRKYs* (12), while chromosome 12, and 5 contained only one *CoWRKY* gene. In addition, five chromosomes (Chr1, 2, 4, 8, 14 and 15) all had 5 *CoWRKY* genes. Chromosome 3, 6, 7, 9, 11, and 13 harbored 7, 2, 7, 3, 8, and 8 *CoWRKYs*, respectively. However, *CoWRKY32* and -*87* mapped onto scaffold_505_fragment_2 and scaffold_290_fragment_25 separate scaffolds and were not assembled into any chromosomes.

**Figure 5:**
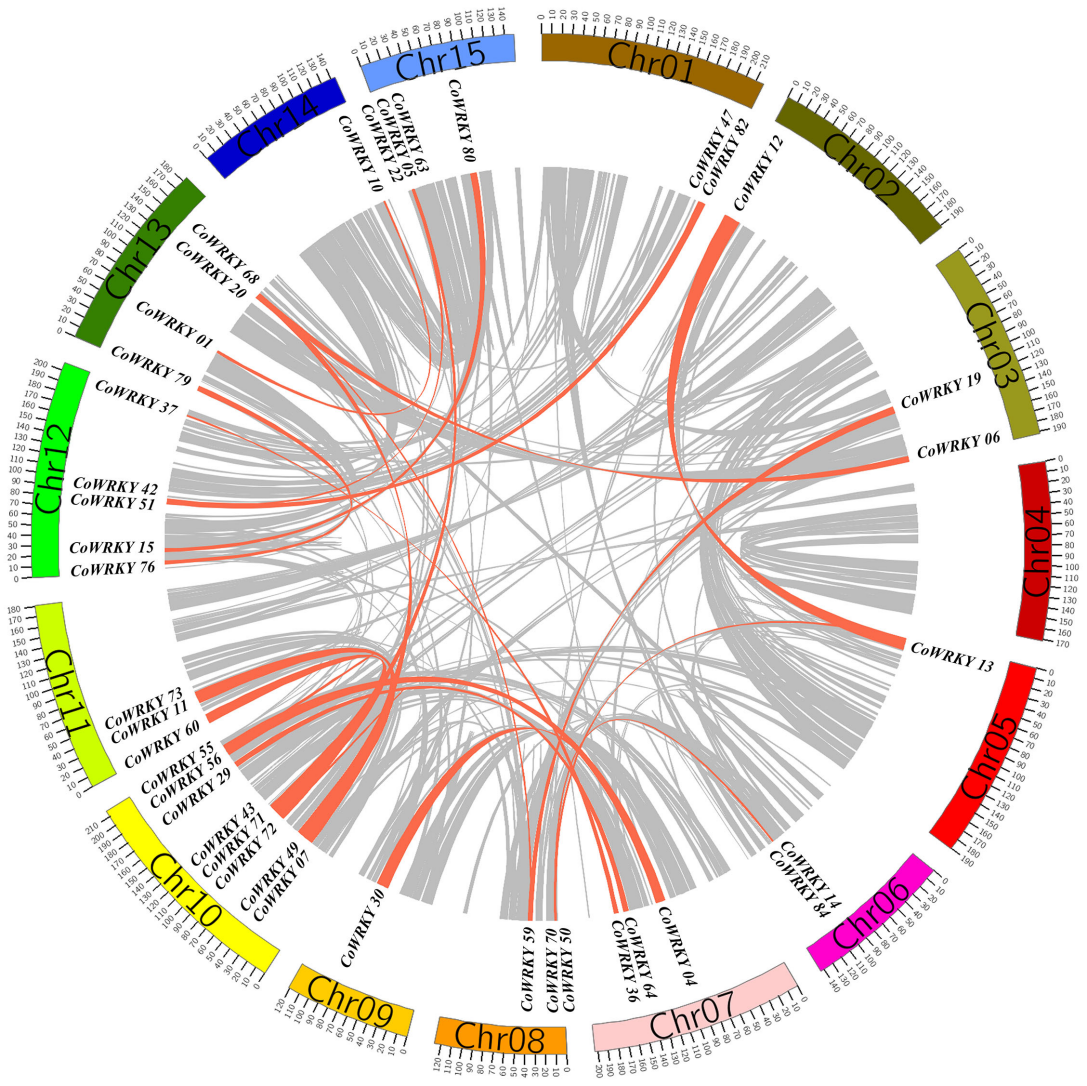
The segmental duplications of *CoWRKY* members. The different color blocks indicated the part of *C. oleifera* chromosomes. The gray lines indicated all segmental duplications in the *C. oleifera* genome, and the red lines indicated segmentally duplicated WRKY gene pairs.

In the evolutionary history of the species, gene tandem duplication and segmental duplication play crucial roles in the generation of the gene family. In general, two or more genes located in the same chromosomal within 200 kb was considered originated from a gene cluster. The gene duplication analysis of *CoWRKYs* indicated that 6 tandem duplication genes were detected to generate three gene clusters (*CoWRKY49/07*, *11/73* and *65/05*), which were distributed in the chromosome 10, 11, and 15, respectively (**Figure 5**). In addition, the segmental duplication events were essential for the evolution of *CoWRKY* gene family. Forty *CoWRKYs*, which were distributed on other 14 chromosomes, could be involved in 28 segmental duplication events.

### Expression patterns of *CoWRKYs* under P deficient stress based on RNA-seq

**Figure 6:**
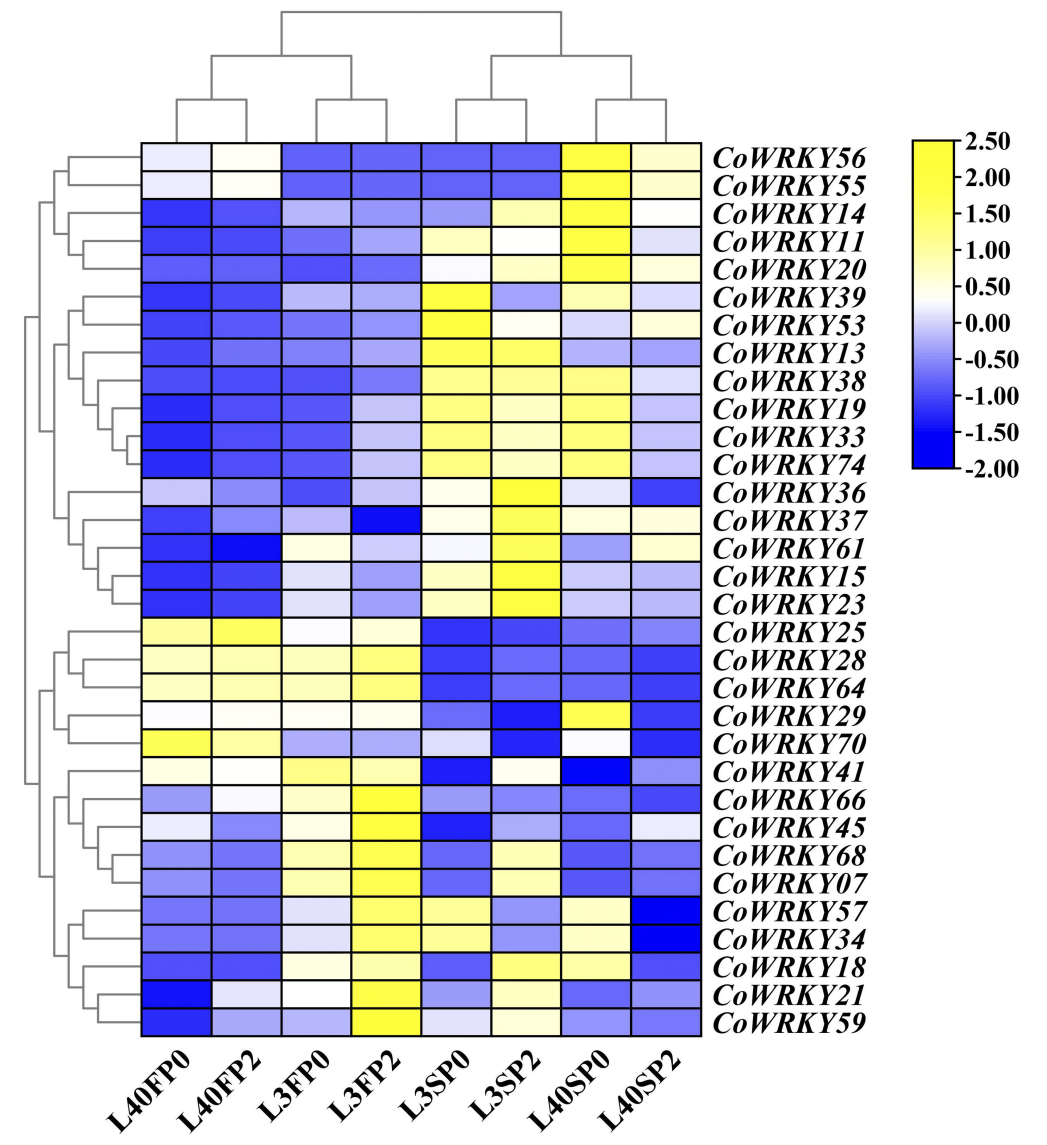
Transcriptomic analysis of the *CoWRKY* genes in response to P deficient stress. Samples were collected from the control and P deficient stress treatment after 30 and 90 days. FP0 and SP0 indicated P deficiency treatment after 30 and 90 days. FP2 and SP2 stood for control treatment after 30 and 90 days. Color scale represented the fold change of P_0_ to control of *CoWRKYs* with normalized log_2_-transformed count value.

To investigate the differentially expression of *CoWRKYs* to the P deficiency stress, the identified 89 *CoWRKYs* were carried based on transcriptome sequence data under the P deficiency treatment. The relative expression levels were determined under control and treatments using two different P-efficiency varieties after 30 and 90 days. The results indicated that 32 differentially expressed *CoWRKY* genes were induced by P deficiency stress (**Figure 6**). Some *WRKY* genes exhibited significant cultivar specificity during P deficiency stress. For instance, *CoWRKY56* and *-55* was highly expressed in the P-efficient variety of CL40, but weakly expressed in the P-inefficient variety of CL3 under the P deficiency treatment. *CoWRKY14, -11, -20, -39, -38, -19, -33*, and -*74* showed relatively higher expression levels under the P deficiency treatments in CL40 after 90 days. *CoWRKY70* differed completely between the two cultivars under both treatments after 30 days. Nonetheless, the expression level of *CoWRKY70* was significantly higher under the P deficiency treatments after 90 days than that of under the control for both cultivars. *CoWRKY29* was highly expressed in CL40 under the P deficiency stress after 90 days, but rarely expressed in CL3. The expression levels of *CoWRKY57* and *-34* were significantly high under the P deficiency treatment after 90 days, but relatively low under the control, especially rarely detected for CL40. According to the classification and the expression levels of *CoWRKYs* under the P deficiency stress, five *CoWRKY* genes, including *CoWRKY11* (Group III), *-14* (Group II-c), *-20* (Group I), *-29* (Group II-d) and *-56* (Group II-b), were selected for subsequent qRT-PCR analysis.

### Expression patterns of *CoWRKY* genes in different tissues

**Figure 7:**
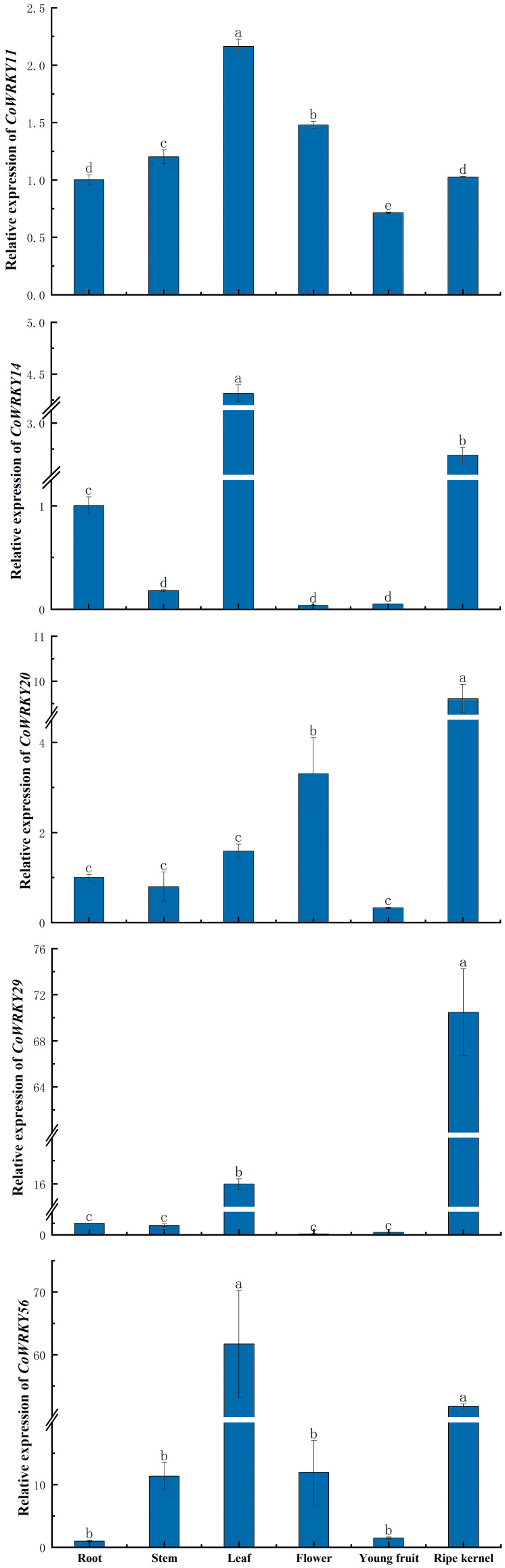
The expression patterns of *CoWRKY* genes in six different tissues. Columns labeled with different letters were significantly different (*p* < 0.05) among different tissues.

The tissue specific expression patterns of 5 selected *CoWRKY* genes were assessed in six tissues, including roots, stems, leaves, flowers, young fruits and ripe kernels of *C. oleifera* trees (**Figure 7**). The expression of different *CoWRKY* genes were involved in different tissues. *CoWRKY14* and *-56* exhibited the highest expression level in leaves followed by in ripe kernels. *CoWRKY2*9 were highly expressed in ripe kernels, of which expression level in ripe kernels was 5 times higher compared to that of in leaves. The expression of *CoWRKY20* performed most in ripe kernels, followed by in flowers. Collectively, the results showed that all five *CoWRKY* genes were highly expressed in leaves but limitedly expressed in roots, stems, and young fruits.

### Expression patterns of *CoWRKY* genes under P deficiency stress based on qRT-PCR

**Figure 8:**
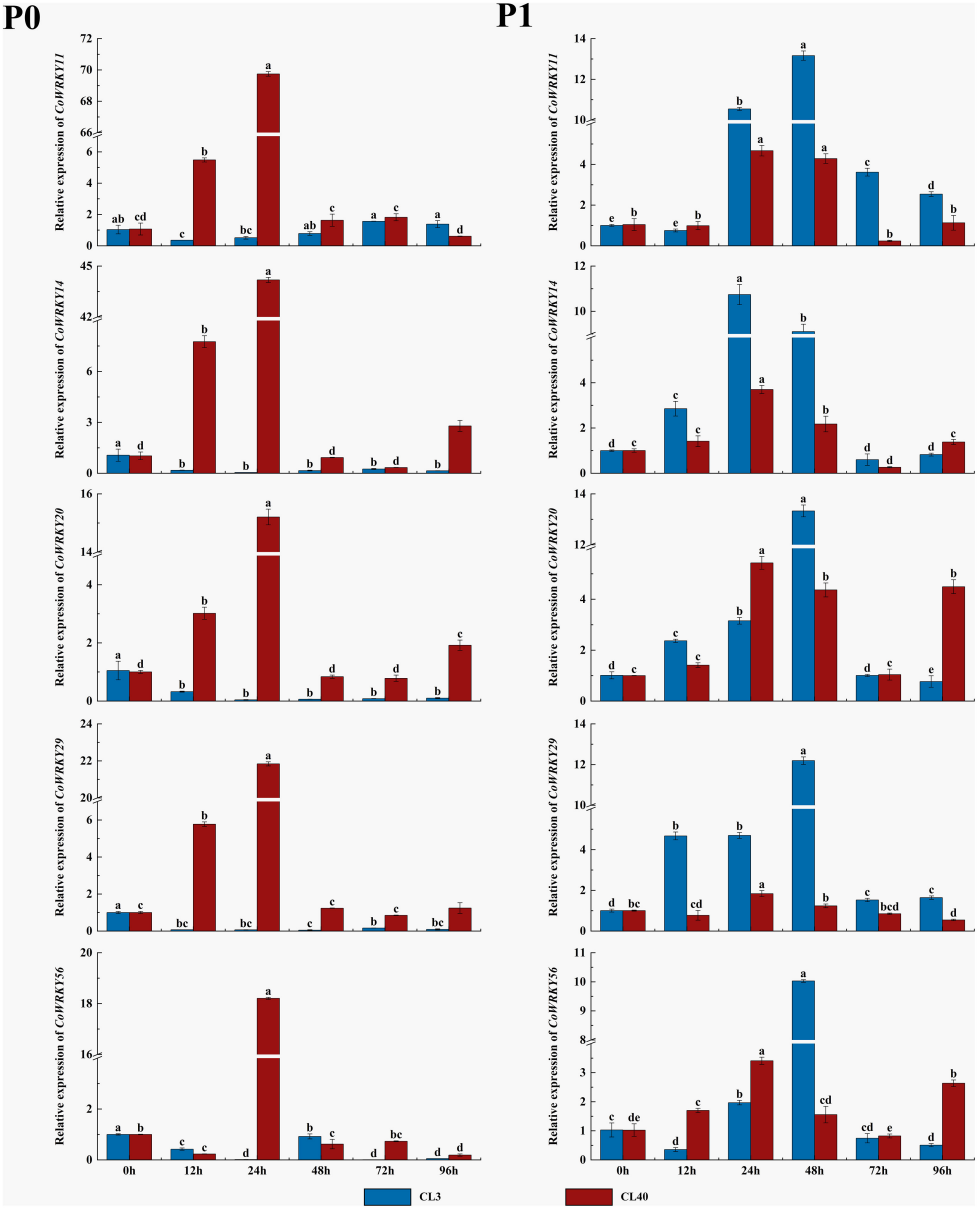
Relative expression of 5 *CoWRKY* genes under P deficient stress. The data were calculated by the 2^−ΔΔCt^ method. The error line represented the standard deviation (n = 3). P_0_ indicated P deficiency treatment, and P_1_ for control treatment. Columns labeled with different letters were significantly different (*p* < 0.05) among the treatment times for each variety.

The expression patterns of five selected *CoWRKY* genes were investigated in P-inefficiency variety of CL3 and P-efficiency variety of CL40 under P deficiency condition (**Figure 8**). The results indicated that all five *CoWRKYs* were upregulated in both cultivars under the control treatment. Furthermore, these five genes exhibited obvious cultivar-specific expression patterns under the P deficiency treatment. They were upregulated in CL40 cultivars, while downregulated in CL3 cultivars. Moreover, five *CoWRKYs* showed similar expression trends in CL40 after P deficiency stress. Their expression levels generally increased initially, reached the peak at 24 h and then decreased. Among them, *CoWRKY11* and -*14* were induced by P deficiency with 70-and 44-fold induction in CL40 cultivars at 24 h, respectively.

**Figure 9:**
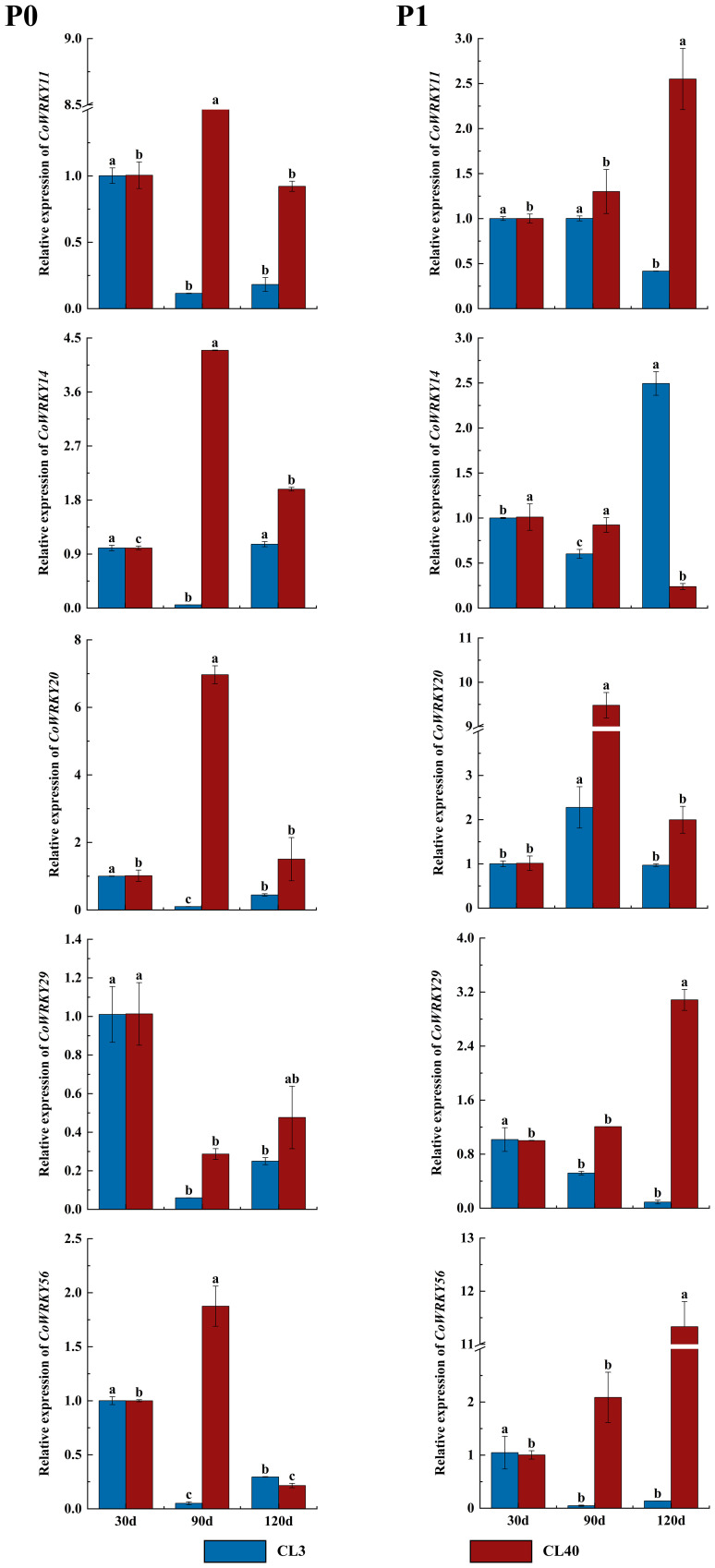
Expression patterns of *CoWRKY* genes under P deficiency during a longer treatment duration. The data were calculated by the 2^−ΔΔCt^ method. The error line represented the standard deviation (n = 3). P_0_ indicated P deficiency treatment, and P_1_ for control treatment. Columns labelled with different letters were significantly different (*p* < 0.05) among the treatment times for each variety.

The expression patterns of *CoWRKYs* were further investigated under P deficiency with a longer treatment duration (**Figure 9**). The result showed similar cultivar-specific expression patterns of five *CoWRKY* genes under the P deficiency treatment, especially treated after 90 d. For example, in CL40, the expression levels of *CoWRKY11*, *-14*, *-20*, and *-56* were upregulated initially and then downregulated after P deficiency treatment, reaching the highest expression level after 90 d. However, the expression of *CoWRKY29* was downregulated firstly and then up-regulated under P deficiency treatment. In CL3, *CoWRKY* genes showed the relative low expression during the entire experiment except *CoWRKY56*.

## Discussion

*C. oleifera* is an hexaploid (2n=6x=90) and mainly planted in acid soil conditions characterized with P deficiency. The shortage of P seriously impacts the production of oil-tea plants and the quality of tea-oil (Wu et al., 2019). The emergence of the genome sequences of the diploid *C. oleifera* var.”*Nanyongensis*” offers an important resource for *C. oleifera* researches. It can also provide the crucial reference to explore genetic basis of functional diversity for *C. oleifera*. WRKY family, as a plant-specific transcription factor, has been demonstrated to participate in plant growth, development and stress responses. With the aim of genetic improvements of agronomically important traits for *C. oleifera*, this systematic analysis identified potential *CoWRKYs* in the diploid *C. oleifera* genome, and examined their expression patterns between different cultivars under P deficiency. This study provided the foundation for further research into the functions of *WRKY* genes in plants.

In this study, a total of 89 *CoWRKY* genes were identified in diploid and wild progenitor of *C. oleifera*, greater than in some woody species such as *C. sinensis* (56) (Wang et al., 2019), *Pinus massoniana* (31) (Yao et al., 2020), *Cunninghamia lanceolate* (44)(Zeng et al., 2019), and *Ginkgo biloba* (40)(Cheng et al., 2019), but fewer than in *Arachis hypogaea* (158, allo-tetraploid) (Zhao et al., 2020), strawberry (222, allo-octoploid) (Zhou et al., 2022), and soybean(174, paleopolyploidy) (Yang et al., 2017). By inference, cultivars of *C. oleifera*, as a hexaploid, were predicted to harbor much larger numbers of WRKY proteins in the genome. In addition, the chromosome distribution and gene duplication analysis indicated that 40 *CoWRKYs* were segmentally duplication genes and involved in 28 segmentally duplication events, suggesting that the majority of *WRKYs* in Camelia oil tree arose from duplication of chromosome regions or the whole chromosomes. The *WRKY* genes after duplication in *C. oleifera* had similar biological functions and were induced by the stresses at the same time, generating a complex regulatory network and participating in the regulation of gene expression.

Phylogenic tree analysis indicated that the identified 89 *CoWRKYs* shared common motifs and divided into three main clades, with Group II further clustering into five subgroups. These results indicated that the CoWRKY proteins were highly conserved and strongly consistent with the group classification of the WRKY family in other higher plants (Rinerson et al., 2015). However, some variants were detected, which was likely related to neofunctionation, subfunctionation and pseudogenization of the *CoWRKY* family (van Verk et al., 2008; Muthamilarasan et al., 2015). For instance, the loss of conserved heptapeptide was observed in Group I (*CoWRKY87*) and subgroup II-c (e.g. the motif of WRKYGKK in *CoWRKY2* and -*3*). It was reported that mutation occurring in members from subgroups II-c was likely involved in changing the DNA biding affinity and resulting in the diversity of binding specificity. In addition, some *CoWRKY* genes were detected to contain flawed C2H zinc finger structure in their conserved domain. Previous research has indicated that the conserved residues of the WRKY domain was required for the proper folding of the DNA-binding zinc finger (Maeo et al., 2001). Mutations in sequences at N-terminal side of zinc finger-liker motif was likely to reduce or abolish the DNA binding activity.

The cis-acting elements in gene promoters influence greatly on the gene expression patterns through the binding sites of transcription factors (Wei et al., 2022). The cis-acting elements analysis of *CoWRKY* genes searched a series of hormone-, development- and stress-related cis-acting elements in the promoter regions of *CoWRKYs.* In general, the WRKY proteins can be regulated by hormones and meanwhile contribute to the regulation of hormone signaling during many biological processes (Rushton et al., 2012). Among nine hormone related cis-acting elements in *CoWRKYs*, the ABRE cis-acting element had the largest number (65) and controlled the synthesis of ABA, which is crucial signal pathway in the mediation of plant resistance. Among 5 stress related cis-acting elements, the WUN-motif cis-acting element contained the largest number (70), which are recognized as wound-responsive elements. Furthermore, LTR, TC-rich repeats and ARE elements involved in abiotic resistance were detected in the *CoWRKYs*. It is speculated that *CoWRKYs* played a crucial role in mediating regulation of stress responses in *C. oleifera*.

P deficiency has a negative effect on plant growth and development of *C. oleifera*, and decreases Camellia oil yield. In this research, 32 *CoWRKY* genes were detected to perform different expression levels in P sufficient and deficient supplies. The expression patterns of some *CoWRKY* genes showed significant cultivar specificity along with the increase of stress duration. For example, *CoWRKY11*, -*14*, *-20*, -*29* and -*56* had higher positive impact on P-efficient CL40 cultivars compared with that on P-inefficient CL3 cultivars (**Figure 8**). Furthermore, the expression of *CoWRKY11* and -*14* were 70-and 44 times higher under P deficiency with induced in CL40 cultivars compared with that in CL3 at 24h, respectively (**Figure 8**). *WRKY* genes in the mediating P deficiency stress responses have been detected in many other species, suggesting the similarities in gene function among homologus genes. For instance, overexpression of *AtWRKY45* (the orthologous gene of *CoWRKY14*) positively regulated the expression of PHT1;1 by binding to the promoter region, improving the inorganic P content of roots under P deficiency and promoting the absorption of inorganic P (Wang et al., 2014). *OsWRKY74*, the orthologous gene of *CoWRKY11*, was significantly induced in the early stage of low-P treatment in roots (Dai et al., 2016).

Many literatures have long demonstrated that large genotypic differences in P efficiency could be mainly attributed to the increased P acquisition efficiency (PAE) characterized with changes in root growth-related phenotypes, including improving root hairs or branching angle of basal roots, when P availability was limited (Wang et al., 2010). This adaptation process has been involved in related gene expression of P uptake and the regulation of transcriptional factors in roots. For example, a recent research on post-transcriptional regulation of microRNA expression in roots under low-P stress in *C. oleifera* indicated that *WRKY53* was one of the three hub transcriptional factors, which were the targets of differentially expressed mRNAs (Chen et al., 2022). However, some researches suggested efficient re-translocation and re-use of the stored P were more important in the breeding or biotechnology of P-efficient varieties, such as the translocation from absorbed P metabolically inactive shoots to active leaves or fruits, or the carbon cost balance between the root development and plant biomass (Wang et al., 2010; Zhang et al., 2017). However, the underlying mechanism was poorly understood. In *C. oleifera*, P efficient variety with greater root-to-shoot ratio had no significant differences in fine roots (less than 1mm in diameter) compared to the P-inefficient varieties under P deficiency (Zeng et al., 2022), but showed relative higher contents of chlorophyll, soluble protein and peroxidase in leaves (Zeng et al., unpublished), indicating that the expression of many genes related to signaling pathways, carbon metabolism and chlorophyllide biosynthesis were affected by P deficiency (Park et al., 2011). WRKY family genes have been reported to play equally important roles in shoots and leaves for P-deficiency tolerance (Kumar et al., 2021). In this study, 32 *WRKY* genes in *C. oleifera* leaves were induced when treated with P deficiency, indicating that *CoWRKYs* played a crucial role in the transportation and recycling P in leaves by affecting diverse metabolic pathways. Furthermore, the underlying mechanism of *WRKY* to participate in the regulation responding to the P deficiency in *C. oleifera* are diverse and complex, especially in P-efficient *C. oleifera* varieties.

## Conclusion

In this study, comprehensive analysis of *CoWRKY* genes and the expression patterns under P deficiency in *C. oleifera* were carried out. According to the diploid genome *C. oleifera*, a total of 89 *CoWRKY* genes was identified and divided into three groups, with group II further classified into five subgroups based on the phylogenetic relationships. Some variants and mutations were detected in the gene structure and conserved domain of *CoWRKYs*, suggesting that neofunctionation, subfunctionation and pseudogenization may occur in the *CoWRKY* family. Segmental duplication events were considered as the primary driver in the expanding process of *WRKY* gene family in *C. oleifera*. *CoWRKY* gene expression indicated that 32 *CoWRKY* participated in the regulation on responses to P deficient stress and shed light on the function of *CoWRKY* genes in *C. oleifera*. The sensitivity of some *CoWRKYs* on the P-efficient variety CL40 clarified the cultivar specificity of *C. oleifera* on P deficiency tolerance. These results will provide a valuable resource for further investigation of functional characterization of *CoWRKY* genes involved in P deficiency stress resistance in *C. oleifera*.

## Data availability statement

The data presented in the study are deposited in NCBI, accession number PRJNA831290.

## Author contributions

WS designed the experiment, processed the data and draft the manuscript; ZZ and JZ prepared the materials and performed the experiment; RC and YZ processed and interpreted the data; DH provided the technical guidance for the experiment; JL conceived the study and revised the manuscript. All authors contributed to the article and approved the submitted version.

## Supplementary material

Supplementary Figure 1 Multiple sequence alignments of WRKY domains in each group of CoWRKY proteins.
