# Supplementary figures and images for: Genome-wide identification of the WRKY gene family in Camellia oleifera and expression analysis under phosphorus deficiency
Source: Front Plant Sci. 2023 May 25;14:1082496. doi: 10.3389/fpls.2023.1082496 (PMC10249505; doi:10.3389/fpls.2023.1082496)

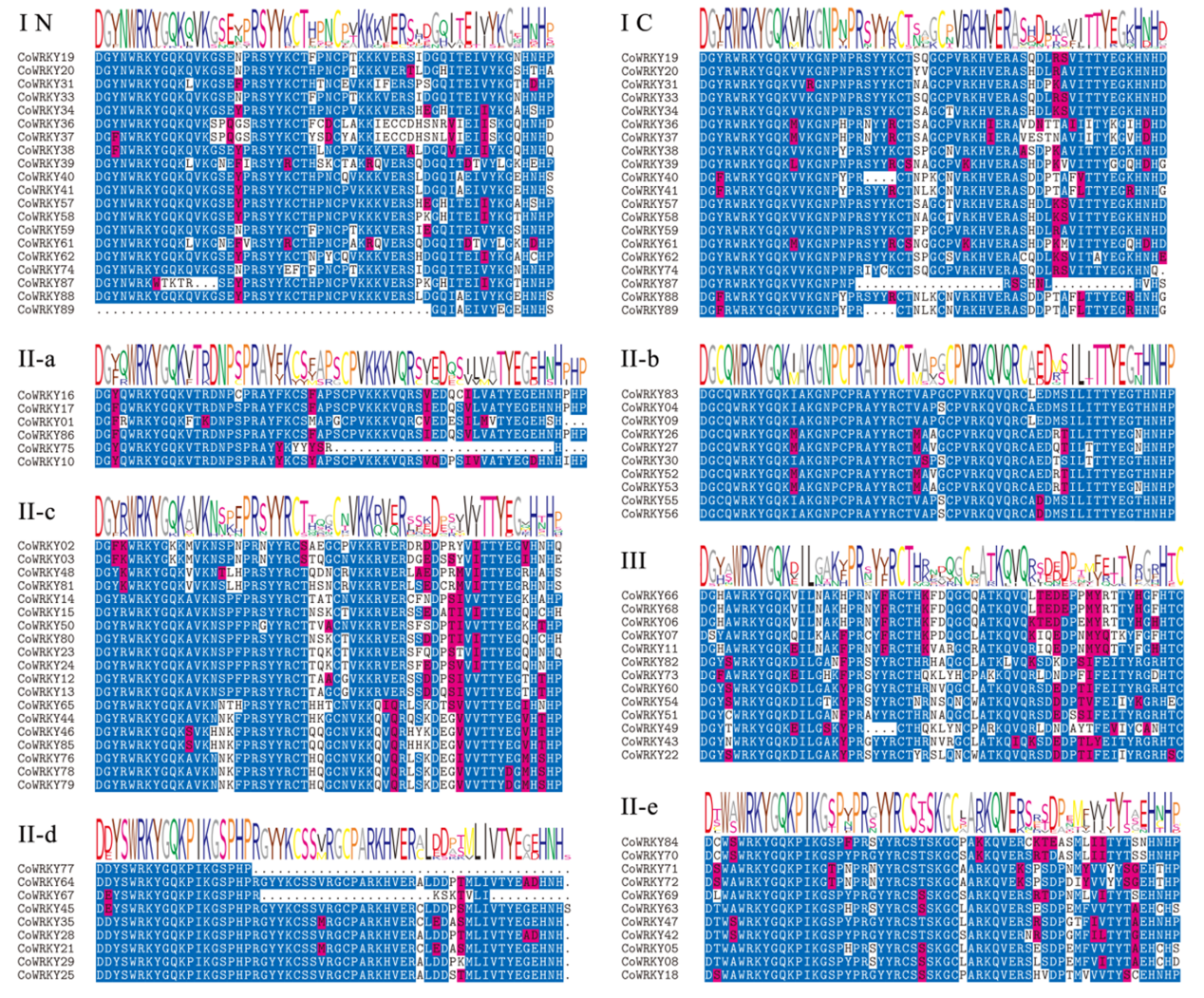

Supplement: Supplementary Figure 1 — Multiple sequence alignments of WRKY domains in each group of CoWRKY proteins. [file Image_1.tif]
